# Detection of Human Traffic Controllers Wearing Construction Workwear via Synthetic Data Generation

**DOI:** 10.3390/s25030816

**Published:** 2025-01-29

**Authors:** Seunghyun Baik, Euntai Kim

**Affiliations:** School of Electrical and Electronic Engineering, Yonsei University, Seoul 03722, Republic of Korea; shbaik104@yonsei.ac.kr

**Keywords:** human traffic controller detection, image synthesis, style and pose customization

## Abstract

Developing Level 3 or higher autonomous vehicles requires the ability to follow human traffic controllers in situations where regular traffic signals are unavailable, such as during construction. However, detecting human traffic controllers at construction sites is challenging due to the lack of dedicated datasets and variations in their appearance. This paper proposes a method for detecting human traffic controllers by generating synthetic images with diffusion models. We introduce a color-boosting technique to enhance image diversity and employ a cut-and-paste mechanism for seamless integration into realistic road scenes. We generate 19,840 synthetic images, combined with 600 real-world images, to train a YOLOv7 model. The trained model achieves an *AP*_50_ score of 73.9%, improving by 32.9% over the baseline. The HTC600 dataset used in our experiments is publicly available to support autonomous driving research.

## 1. Introduction

Autonomous vehicles are advancing to Level 3 and beyond. As they progress, they face increasingly complex and unpredictable road conditions, including construction zones. In these scenarios, vehicles cannot rely solely on static traffic signs or digital maps. They must detect human traffic controllers and interpret their gestures to navigate safely, as these controllers play a vital role in managing traffic flow. Failure to detect them can cause serious accidents. People might think that with recent advances in object detection, such as YOLO [1] and DETR-based approaches [2,3], detecting human traffic controllers would be straightforward. However, this is not the case. The main challenge in training human traffic controller detectors is the lack of dedicated datasets. Addressing this challenge is complicated by the diverse appearances of human traffic controllers. These include variations in workwear and helmet colors, as well as a wide range of signaling poses. Overcoming these challenges requires innovative approaches to data generation and augmentation.

Recent advancements in synthetic data generation have demonstrated their potential to tackle such data scarcity issues across various domains. Leveraging generative AI techniques, such as diffusion-based methods and AI-generated content, researchers have achieved significant improvements in object detection by creating diverse and realistic datasets [4,5,6,7,8]. Inspired by these advancements, this research aims to synthetically generate high-quality images of human traffic controllers with diverse workwear and poses. To address this problem specifically, we build on the synthetic data generation techniques that have been proven valuable in other domains [9,10,11,12] where synthetic data generation and enhancement techniques have been proven valuable.

**Figure 1:**
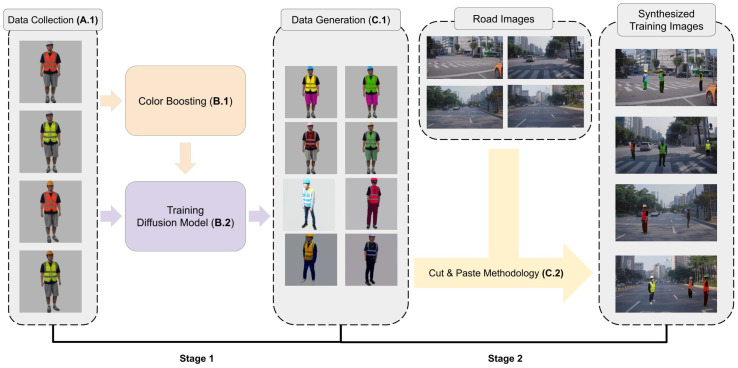
Overview of the proposed method: Generating diverse human traffic controller images from base images and integrating them with road images using a cut-and-paste mechanism to create realistic synthesized images.

The overall process consists of two main stages, as shown in Figure 1. Before starting these stages, we collect 600 real images of human traffic controllers and build a dataset named HTC600 (Human Traffic Controller dataset with 600 images) (A.1). In the first stage, we train a person image synthesis network, PIDM [13], using HTC600 and the DeepFashion dataset [14] (B.2). To further enhance the model’s ability to produce diverse outputs, we produce a technique called color boosting for image synthesis (B.1). This technique diversifies the colors of workwear items such as helmets, vests, and pants, ensuring that the generated images effectively capture the wide range of visual characteristics associated with human traffic controllers.

In the second stage, we generate a synthetic human traffic controller using PIDM (C.1) and integrate the generated human traffic controller images into realistic road construction environments using a cut-and-paste methodology (C.2) inspired by prior works [9,10,11,12]. This approach places human traffic controllers in realistic positions and resizes them based on their scene placement, blending them seamlessly with the background. This step is critical for creating training images that not only include diverse human traffic controller appearances but also represent realistic scenarios in which human traffic controllers are encountered on roadways.

To evaluate the effectiveness of our approach, we built the HTC600 dataset, which consists of 600 real images for training and 135 real-world images for testing. The datasets include images captured in both controlled and uncontrolled environments, as well as images sourced from the Internet. This diverse dataset provides a robust foundation for benchmarking human traffic controller detection performance. The HTC600 dataset is publicly available for download at https://github.com/SeungHyun104/HTC600 (accessed on 16 December 2024).

In the experiment, we synthesized a large number of training datasets using only 600 human traffic controller images. This approach achieved a robust result with a *AP*_50_ score of 73.9% on the HTC600 test dataset.

In summary, this work makes the following contributions:

- We present the first study addressing human traffic controller detection through the generation of synthetic images.
- We propose a novel image synthesis framework that combines advanced techniques, such as color boosting and perspective-aware cut-and-paste integration, to generate diverse and realistic datasets from limited initial data, specifically tailored for robust human traffic controller detection.
- We develop and publicly release the HTC600 dataset, comprising both real-world and synthetic images, to address data scarcity and support further research in human traffic controller detection.
- By leveraging the synthesized dataset, we achieve a significant improvement in detection performance, as evidenced by an increase in *AP*_50_ of 32.9% compared to the baseline.

Our experimental results confirm that using the synthesized dataset substantially improves the robustness and accuracy of human traffic controller detection models. This advancement is a critical step towards enhancing the safety and reliability of autonomous driving systems as they navigate the complexities of real-world road construction environments.

## 2. Related Works

### 2.1. Worker Detection

Worker detection in construction sites is important, especially with the rise of automated safety systems and autonomous vehicles. These tasks are generally approached as part of object detection, with specific adaptations for construction environments. Traditional methods often use models like YOLO [1]. These models include features like enhanced feature extraction and attention mechanisms to manage visually challenging scenarios. For example, Son et al. [15] trained YOLOv4 using large public datasets like ImageNet [16] and Pascal VOC [17], along with a construction site dataset of 22,000 images, to achieve robust worker detection.

Recent advancements have focused on addressing challenges such as occlusion, overlapping individuals, and varied worker postures. Park et al. [18] introduced SOC-YOLO, an adaptation of YOLOv5, which incorporated improvements such as a distance intersection over union (DIoU) loss function and a weighted-triplet attention mechanism. These modifications significantly enhanced detection accuracy for partially occluded workers in crowded environments, proving valuable for safety monitoring in complex construction settings.

### 2.2. Pose-Guided Person Image Synthesis

Pose-guided person image synthesis has seen significant advancements, especially with the introduction of various models aimed at generating human images in desired poses. Early methods, like Ma et al. [19], took a coarse-to-fine approach but often faced issues with feature misalignment due to the simple concatenation of source images and poses. To improve this, Esser et al. [20] separated appearance and pose using a VAE-based framework, introducing skip connections for better alignment. Siarohin et al. [21] refined this approach by employing deformable skip connections to handle local spatial transformations. Meanwhile, Ren et al. [22] developed GFLA, which used flow fields to warp image patches and improve pose alignment. Models like ADGAN [23] and PISE [24] introduced texture encoding and parsing maps to enhance the quality of synthesized images.

More recently, Bhunia et al. [13] proposed a diffusion-based model called PIDM. This model departs from traditional GAN-based approaches.

Instead of attempting complex pose transformations in a single pass, PIDM breaks the process into simpler conditional denoising steps, preserving appearance details more effectively. Additionally, PIDM uses disentangled classifier-free guidance to ensure the output image closely matches both the source image’s style and the target pose, even in complex scenarios.

In our work, we adopt PIDM for pose-guided person image synthesis, leveraging its diffusion-based approach to generate realistic images while maintaining high-quality appearance details, even under challenging pose variations.

### 2.3. Synthetic Data Generation for Object Detection

Creating synthetic training data is essential for addressing the limitations of real-world datasets. This is particularly important in object detection and other machine learning tasks. Synthetic data generation techniques allow researchers to create diverse datasets that would be challenging or costly to collect in real-world settings. Recent advancements have focused on domain-specific applications, leveraging methods that ensure both realism and variability in the generated datasets.

Recently, diffusion-based models and generative AI play key roles in this field. Reutov et al. [4] leveraged diffusion models to produce urban traffic datasets for vehicle detection, while Zhu et al. [5] introduced ODGEN for synthetic data generation in specialized object detection tasks, demonstrating substantial improvements in model robustness. Likewise, Chen et al. [6] developed RailFOD23 for detecting anomalies on railway transmission lines, showcasing how generative AI can boost detection accuracy.

In addition, other methods have proven effective in enhancing the adaptability of synthetic datasets across various domains. Oric et al. [7] added diverse environmental variables to synthetic vehicle detection datasets, and Goyal and Mahmoud [8] surveyed advanced techniques to highlight the importance of realism and variability. In construction-related detection, Seong et al. [11] introduced a content-swapping technique that decomposed and recombined sign components to mitigate data scarcity and improve model accuracy. Similarly, Lee et al. [12] developed an image synthesis framework leveraging domain transfer and blending techniques, effectively addressing data scarcity for fallen person detection and enhancing detection performance across diverse road scenarios.

Building on these advancements, our work adopts a cut-and-paste methodology but incorporates novel enhancements tailored to human traffic controller detection. Specifically, we integrate a color boosting method and the PIDM model [13] to generate a diverse and comprehensive synthetic dataset. The color boosting method utilizes Grounding DINO [25] and SAM2 [26] models to modify human traffic controller attire colors, creating variability while maintaining dataset integrity. The PIDM model further complements this by generating realistic images with varied poses and styles, addressing the challenges of pose diversity and appearance variability.

By combining these techniques, our approach creates a robust synthetic dataset that captures the complex visual characteristics of human traffic controllers in road construction environments. This dataset not only alleviates the challenges of collecting sufficient real-world data but also enhances the robustness and accuracy of detection models in scenarios where diverse appearances and poses of human traffic controllers are critical. Our work specifically addresses the unique challenges of detecting human traffic controllers in dynamic road conditions, bridging the gap left by traditional worker detection approaches.

### 2.4. Model Compression

As deep learning models continue to grow in size and complexity, they often face deployment challenges on resource-constrained devices. Model compression techniques, such as pruning and quantization, have emerged as effective solutions to reduce both computational demands and memory usage while still maintaining acceptable levels of performance.

Pruning involves removing unnecessary weights or channels to yield a more compact and efficient network structure. For instance, Fang et al. propose DepGraph [27], a framework that generalizes structural pruning across various neural network architectures by analyzing dependencies between layers.

Quantization, on the other hand, reduces the numerical precision of weights and activations. This approach can significantly decrease model size and inference latency, particularly on specialized hardware. Günay et al. present LPYOLO, a low-precision variant of the YOLO framework tailored for face detection on FPGA devices [28]. Their experiments show that carefully quantized object detection networks can achieve near-real-time performance with a notable reduction in power consumption.

Overall, pruning and quantization are complementary strategies for model compression: pruning effectively removes redundant network components, while quantization optimizes numerical representation. Together, they allow deep models to run efficiently on hardware with limited resources, providing a promising path forward in scenarios where both latency and energy efficiency are paramount.

In our own work, we also incorporate quantization into YOLOv7 to further reduce computational overhead and facilitate real-time deployment on resource-constrained devices.

## 3. Methods

### 3.1. Overview

**Figure 2:**
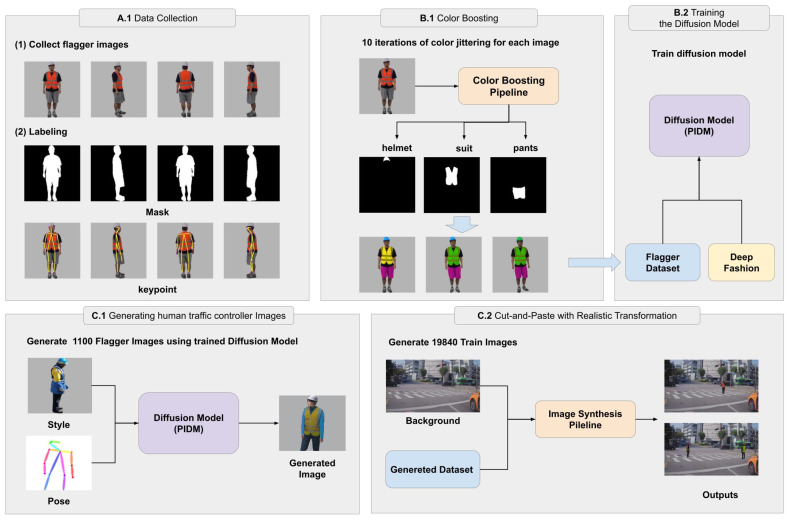
Detailed stage-by-stage overview of the proposed method: The entire process is divided into five steps. (**A.1**) Collecting human traffic controller images and annotating masks and keypoints. (**B.1**) Applying color boosting to diversify human traffic controller appearances. (**B.2**) Training the diffusion model with the augmented human traffic controller and DeepFashion datasets. (**C.1**) Generating diverse human traffic controller images with specified poses and styles using the trained diffusion model. (**C.2**) Integrating the generated images with road backgrounds using a cut-and-paste mechanism with realistic transformations to create a large synthesized training dataset.

The two stages shown in Figure 1 are further divided into five key steps, as illustrated in Figure 2. These five steps can be summarized as follows:

- **Data Collection (A.1)**: We capture human traffic controller images featuring six different individuals. We took the images indoors from various angles, with subjects in a few fixed postures to maximize variability while using a small dataset. We annotated the images with keypoints and masks to support subsequent processing.
- **Color Boosting (B.1)**: A two-stage color boosting process applies to diversify the appearance of human traffic controller images. This process targets specific body parts, such as helmets, suits, and pants, to introduce color variations while preserving dataset integrity.
- **Training the Diffusion Model (B.2)**: The diffusion model PIDM trains using the augmented human traffic controller dataset and the DeepFashion dataset. This enables the generation of realistic images with diverse poses and styles.
- **Generating Human Traffic Controller Images (C.1)**: Using the trained diffusion model, we synthesize a large set of high-quality human traffic controller images with specified poses and styles, ensuring broad variability in the generated dataset.
- **Cut-and-Paste with Realistic Transformations (C.2)**: Human traffic controller images seamlessly integrate into road background images using a cut-and-paste method. Realistic transformations, including perspective-based instance size adjustments, color harmonization, and multi-instance integration, apply to ensure the composites reflect real-world environments.

For a clearer explanation, we provide a pseudo-code of the proposed method in Algorithm 1. Each step in Algorithm 1 matches Figure 2. In the following subsections, we describe the details of each step.
**Algorithm 1** Pseudo-code of the proposed method**A.1:** **Data Collection**1:   Collect human traffic controller images $I_{HTC}$2:   Generate keypoints ($K_{HTC}$) and masks ($M_{HTC}$) of collected images.⁡**B.1:** **Color Boosting**3:Generate masks of each body part ($M_{p}$, $M_{h}$, $M_{v}$) using Ground DINO and SAM2.4:Sample brightness ($b_{i}∼U\left(0.5,1.5\right)$), contrast ($c_{i}∼U\left(0.6,1.4\right)$), saturation ($s_{i}∼U\left(0,2.0\right)$), and hue ($h_{i}∼U\left(-0.4,0.4\right)$).5:Apply color adjustments:6:   $I_{i}^{\prime }=I\cdot \mathrm{AdjustBrightness}\left(b_{i}\right)\cdot \mathrm{AdjustContrast}\left(c_{i}\right)\cdot \mathrm{AdjustSaturation}\left(s_{i}\right)\cdot \mathrm{AdjustHue}\left(h_{i}\right)$.7:Combine adjusted regions and background:8:   $I_{\mathrm{boosted}}=I_{p}^{\prime }⊙M_{p}+I_{h}^{\prime }⊙M_{h}+I_{v}^{\prime }⊙M_{v}+I⊙\left(1-\left(M_{p}+M_{h}+M_{v}\right)\right)$.⁡**B.2:** **Training the Diffusion Model**9:Train PIDM model with Collected and Color Boosted Datasets.**Input:** $I_{\mathrm{DeepFastion}}$, $I_{\mathrm{boosted}}$, $I_{HTC}$, $K_{HTC}$⁡**C.1:** **Generating Human Traffic Controller Images**10:Collect target style human traffic controller images $S_{target}$11:Generate target keypoints $K_{target}$12:Generate human traffic controller image $I_{\mathrm{generated}}$ with target pose $K_{target}$ and style $K_{target}$.**Input:** $S_{target}$, $K_{target}$**Output:** $I_{\mathrm{generated}}$⁡**C.2:** **Cut-and-Paste with Realistic Transformations**13:Collect road background images $I_{background}$14:Identify pasteable regions $M_{pasteable}$ in $I_{background}$.15:Compute vanishing point $y_{max}$ and adjust human traffic controller size ($H^{\prime }$) according to where to attach *y*:16:     $H^{\prime }=H_{\mathrm{base}}\cdot \left(1-\frac{y}{y_{\max}}\right)$.17:Apply cut-and-paste to integrate $I_{HTC}$, $I_{\mathrm{boosted}}$, $I_{\mathrm{generated}}$, into $I_{background}$.18:Harmonize colors between the pasted instance and background ($I_{background}$).**Output:** Final synthesized dataset ($I_{\mathrm{synthetic}}$).

### 3.2. Data Collection

**Figure 3:**
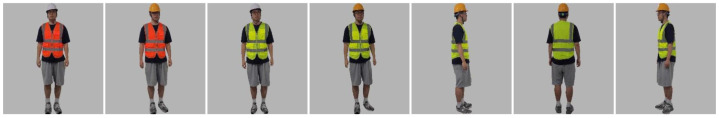
The example of collected human traffic controller images.

As shown in Figure 3, we collected 600 images of human traffic controllers, featuring five different individuals. The five individuals wear four distinct combinations of white and yellow helmets with orange and yellow construction workwear suits. The images are taken from various angles, with individuals posed in a few fixed postures to efficiently generate diverse training datasets.

For labeling, we utilize pre-trained models to streamline the process. Specifically, we use the YOLOv7-pose model [29] for keypoint labeling and the Segment Anything 2 model [26] for mask labeling. This ensures accurate and efficient annotation of both keypoints and masks across the dataset, facilitating further analysis and model training.

### 3.3. Color Boosting

**Figure 4:**
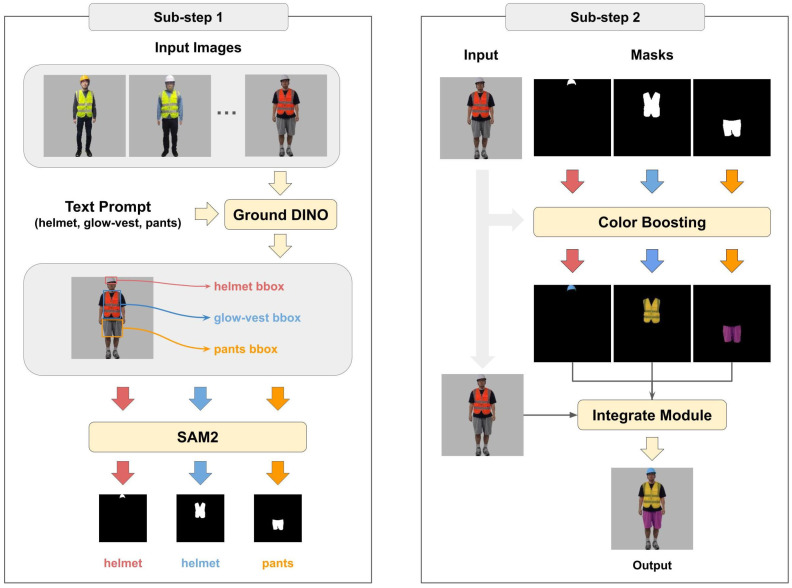
Color boosting process. In sub-step 1, Ground DINO and SAM2 generate masks for specific body parts. In sub-step 2, custom color boosting is applied to these regions to create diverse color variations.

To enhance data variability, we develop a color boosting method consisting of two sub-steps, as illustrated in Figure 4. This approach targets specific body parts, such as the helmet, construction workwear suit, and pants, to introduce diverse color variations while maintaining dataset integrity.

#### 3.3.1. Sub-Step 1: Mask Generation

We employ Ground DINO [25] and SAM2 [26] models to generate masks for each body part. Ground DINO uses text prompts to identify bounding boxes for the helmet, glow-vest, and pants, which are then passed to SAM2 to produce detailed masks. The mask for each region is defined as:

(1) $$M_{i}\left(x,y\right)=\left\{\begin{array}{cc} 1 & \mathrm{if}\;\mathrm{pixel}\;(x,y)\;\mathrm{belongs}\;\mathrm{to}\;\mathrm{region}\;i,\\ 0 & \mathrm{otherwise}, \end{array}\right.$$

where i∈{p,h,v} represents the pants, helmet, and glow-vest regions, respectively.

#### 3.3.2. Sub-Step 2: Color Boosting

To introduce diverse color variations, the brightness ($b_{i}$), contrast ($c_{i}$), saturation ($s_{i}$), and hue ($h_{i}$) for each region are independently adjusted. These attributes are sampled from uniform distributions as follows:

(2) $$\begin{array}{cccccccc} b_{i} & ∼U(0.5,1.5), & c_{i} & ∼U(0.6,1.4), & s_{i} & ∼U(0,2.0), & h_{i} & ∼U(-0.4,0.4), \end{array}$$

where i∈{p,h,v} represents pants, helmet, and glow-vest regions. The color-adjusted regions are computed as:

(3) $$I_{i}^{\prime }=I\cdot \mathrm{AdjustBrightness}\left(b_{i}\right)\cdot \mathrm{AdjustContrast}\left(c_{i}\right)\cdot \mathrm{AdjustSaturation}\left(s_{i}\right)\cdot \mathrm{AdjustHue}\left(h_{i}\right).$$

To apply these adjustments to specific body parts, we use masks generated in Sub-step 1. Each body part is extracted using its corresponding mask:

(4) $$\begin{array}{cccccccc} I_{p} & =I_{p}^{\prime }⊙M_{p}, & I_{h} & =I_{h}^{\prime }⊙M_{h}, & I_{v} & =I_{v}^{\prime }⊙M_{v}, & I_{\mathrm{bg}} & =I⊙\left(1-(M_{p}+M_{h}+M_{v})\right), \end{array}$$

where $I_{\mathrm{bg}}$ represents the background region excluding the masked parts.

Finally, the adjusted components are combined to produce the final image:

(5) $$I_{\mathrm{final}}=I_{p}+I_{h}+I_{v}+I_{\mathrm{bg}}.$$

This process is repeated 10 times per image, introducing various variations in the appearances of human traffic controllers.

**Figure 5:**
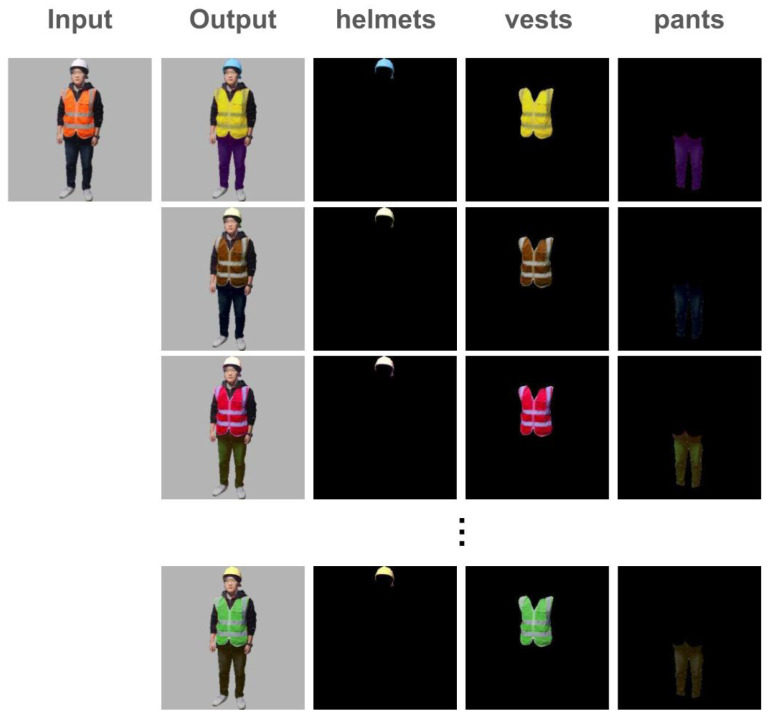
Examples of color boosted human traffic controller images. The generated masks for helmets, vests, and pants accurately correspond to the specified test prompts, as seen in the mask images. The color variations applied to each region demonstrate successful adjustments, showcasing diverse brightness, contrast, saturation, and hue changes while preserving the structural integrity of the original image.

Figure 5 shows the transformation achieved by color boosting. The input image represents the original human traffic controller, while the output images demonstrate diverse variations in the color attributes of helmets, glow-vests, and pants. The corresponding mask images highlight the regions targeted for adjustment.

The method introduces realistic variability in brightness, contrast, saturation, and hue, ensuring that only the intended regions are modified. This targeted approach maintains the structural integrity of the original image while simulating diverse environmental conditions. By generating visually diverse samples, the color boosting process enhances the robustness of the dataset, enabling the detection model to better handle variations in workwear and lighting conditions encountered in real-world scenarios.

### 3.4. Training the Diffusion Model

We train the PIDM [13] model using the collected and color-boosted human traffic controller images, combined with the DeepFashion dataset [14], to generate high-quality images with diverse poses and styles. The overall training process follows the methodology outlined in the original work [13].

The generative modeling process is based on the Denoising Diffusion Probabilistic Model (DDPM), which progressively adds Gaussian noise to the input data during the forward diffusion process, transforming it into pure noise. The forward process is defined as:

(6) $$q\left(y_{t}|y_{t-1}\right)=N\left(y_{t};\sqrt{\alpha_{t}}y_{t-1},\left(1-\alpha_{t}\right)I\right),$$

where $y_{t}$ is the noisy data at time step *t*, $\alpha_{t}$ is a noise scheduling coefficient, and I is the identity matrix. At each step, Gaussian noise ϵ∼N(0,I) is added, perturbing the original data $y_{0}$ into noise $y_{T}$ over *T* timesteps. The resulting noisy data $y_{t}$ at any step can be expressed directly as:

(7) $$y_{t}=\sqrt{{\bar{\alpha }}_{t}}y_{0}+\sqrt{1-{\bar{\alpha }}_{t}}\epsilon ,$$

where ${\bar{\alpha }}_{t}=\prod_{i=1}^{t}\alpha_{i}$ is the cumulative noise scheduling coefficient.

The reverse process reconstructs the original data by iteratively removing the added noise. It is parameterized as:

(8) $$p_{\theta }\left(y_{t-1}|y_{t},x_{s},x_{p}\right)=N\left(y_{t-1};\mu_{\theta }\left(y_{t},t,x_{s},x_{p}\right),\Sigma_{\theta }\left(y_{t},t,x_{s},x_{p}\right)\right),$$

where $x_{s}$ is the target style image, $x_{p}$ is the target pose, and $\mu_{\theta }$, $\Sigma_{\theta }$ are the predicted mean and variance.

During training, the model learns to predict the added noise $\epsilon_{\theta }$ at each step, minimizing the difference between the actual added noise ϵ and the predicted noise $\epsilon_{\theta }$. This is achieved by optimizing the simplified loss function:

(9) $$L_{\mathrm{mse}}=E_{y_{0},\epsilon ,t}\left[∥\epsilon -\epsilon_{\theta }\left(y_{t},t,x_{s},x_{p}\right){∥}_{2}^{2}\right],$$

where ϵ is the actual noise added during the forward process, and $\epsilon_{\theta }$ is the noise predicted by the model. This ensures accurate denoising during the reverse process.

To enhance the stability and quality of training, the hybrid loss function used in [13] combines this Mean Squared Error (MSE) loss with a variance improvement loss:

(10) $$L_{\mathrm{hybrid}}=L_{\mathrm{mse}}+L_{\mathrm{vib}}.$$

Here, $L_{\mathrm{vib}}$ improves the model’s variance prediction $\Sigma_{\theta }$, helping to stabilize the denoising process and refine the quality of the generated images.

By explicitly modeling the relationship between the added noise ϵ and the predicted noise $\epsilon_{\theta }$, the training process enables the model to reconstruct realistic images from noisy inputs. These iterative forward and reverse steps allow PIDM to achieve robust transformations, resulting in high-quality image generation with accurate poses and styles.

### 3.5. Generating Human Traffic Controller Images

Using the trained PIDM model, we generate human traffic controller images to create a robust and diverse dataset. For the generation process, we utilize 50 distinct poses and 22 different styles, resulting in a total of 1100 human traffic controller images (50 poses × 22 styles). These images were designed to include various combinations of styles and poses, ensuring a wide range of variability.

**Figure 6:**
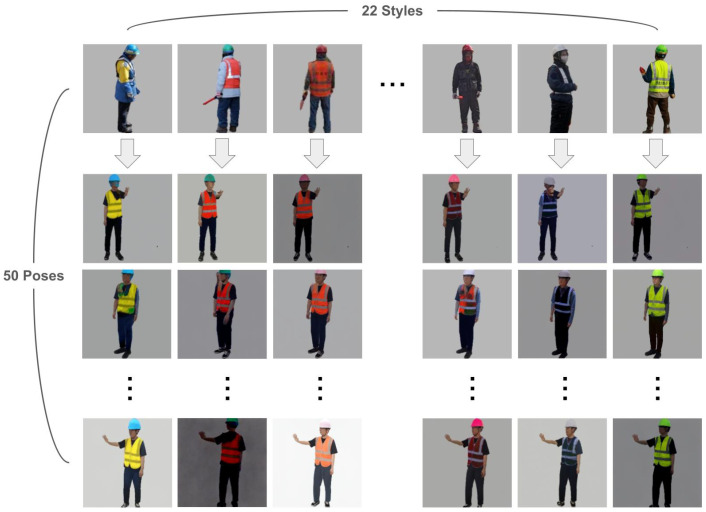
Examples of generated human traffic controller images. The human traffic controller images are created according to the desired styles and poses.

Figure 6 illustrates examples of human traffic controller images synthesized using the PIDM model. These images show the successful application of diverse styles and poses, capturing a wide range of variations in workwear and signaling gestures.

The ability to generate visually realistic and diverse images highlights the effectiveness of the PIDM model in preserving key visual details, such as helmet designs, glow-vest patterns, and body postures. By accurately synthesizing these characteristics, the model significantly enriches the dataset and addresses the challenges posed by the limited availability of real-world data. This diverse data generation improves the robustness of the detection model by providing comprehensive examples that simulate real-world conditions. The synthesized images expand the model’s capacity to handle variability in human traffic controller appearances, thereby improving detection performance in dynamic and complex environments.

### 3.6. Cut-and-Paste with Realistic Transformations

To synthesize realistic training datasets, we employ a cut-and-paste method [9,12,30,31] enhanced with realistic transformations. This process is further divided into three sub-steps: (1) identification of pasteable regions, (2) perspective-based instance size adjustments, (3) multi-instance integration and color adjustments. These sub-steps are designed to seamlessly integrate human traffic controller images into road backgrounds, ensuring that the synthesized images appear natural and realistic.

#### 3.6.1. Pasteable Region Identification

This sub-step is based on our previous work [11]. We first identify pasteable regions as drivable areas in the road background images. These regions were detected using a pre-trained segmentation model, ensuring that human traffic controller images were placed only in feasible locations. The road background images are also taken from [11], which released 992 images collected in Seoul, South Korea.

#### 3.6.2. Instance Size Adjustment Using the Vanishing Point

We develop an instance size adjustment method to paste a human traffic controller into a background image. This sub-step distinguishes the paste in this paper from the one in our previous work [11]. In that work, we used *simple image projection* to paste a road construction sign that appeared below the camera level, whereas, in this paper, we use the vanishing point to paste a human traffic controller shown at or above the camera level. The size adjustment method used here is inspired by [30,32].

Specifically, we use the vanishing point to adjust the size of each human traffic controller based on its vertical position (*y*) in the scene. The base height ($H_{\mathrm{base}}$) of the human traffic controller is set to 750 pixels, representing the object’s height when positioned at the bottom of the image (y=0), but this value can vary depending on the background image height. The vanishing point ($y_{\max}$) corresponds to the biggest *y*-coordinate within the drivable region, as determined by a pre-trained segmentation model. For $0<y<y_{\max}$, the size of the human traffic controller ($H^{\prime }$) is computed as:

(11) $$H^{\prime }=H_{\mathrm{base}}\cdot \left(1-\frac{y}{y_{\max}}\right),\;0<y<y_{\max},$$

where

- $H_{\mathrm{base}}$: The base height of the human traffic controller image in pixels.
- *y*: The vertical position of the human traffic controller in the background image.
- $y_{\max}$: The largest *y*-coordinate within the drivable region, representing the vanishing point.

This equation ensures that the height of the human traffic controller decreases proportionally with its vertical position *y*, maintaining realism by scaling according to the perspective effect, while ensuring $H^{\prime }>0$.

**Figure 7:**
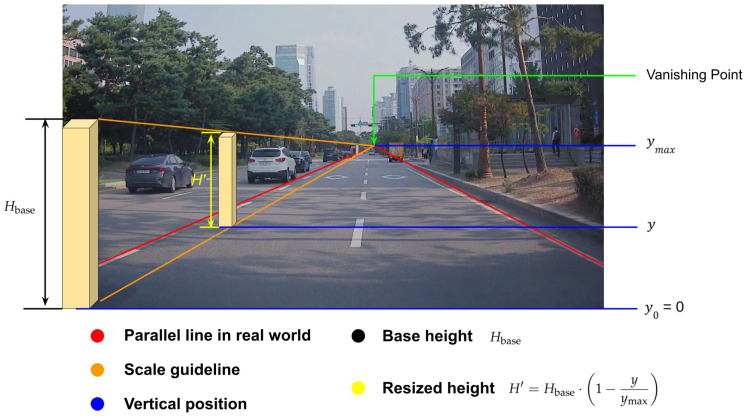
Illustration of the vanishing point-based instance size adjustment. The figure demonstrates how objects positioned higher in the image (closer to the vanishing point) are scaled down to reflect the real-world perspective. The base height ($H_{\mathrm{base}}$) represents the object’s original size at y=0, and the resized height ($H^{\prime }$) adapts according to the vertical position (*y*) and maximum visible *y*-coordinate ($y_{\max}$). Two red lines converge at the vanishing point, while scale guidelines in orange indicate the progressive reduction in object size.

This approach ensures that objects placed higher in the image appear smaller, accurately reflecting the perspective effect. Figure 7 illustrates this process, showing how the human traffic controller size changes based on its position relative to the vanishing point.

#### 3.6.3. Multi-Instance Integration and Color Adjustments

For each road background, we generate up to three instances of human traffic controllers per image, each subjected to independent size adjustment. This allows us to simulate more complex and realistic scenarios. By varying the number and position of human traffic controllers, we ensure a diverse set of training images.

In addition, to enhance the realism of the synthesized images, we apply color adjustment techniques that harmonize the appearance of the pasted human traffic controller instances with the background. This approach takes advantage of the color difference method introduced in our previous work [11]. By aligning the brightness, contrast, and overall tone of the human traffic controller images to match the road background, we minimized visual discrepancies and ensured seamless integration between the pasted objects and their surroundings.

#### 3.6.4. Dataset Statistics

Using this approach, we synthesize a total of 19,840 training images per generation by combining human traffic controller instances with 992 road background images. For each road background, we generate 20 synthetic variations, ensuring a diverse and representative dataset. These realistic synthetic images significantly enhance the robustness of our detection model by providing a wide range of scenarios for training.

In particular, we mitigate the risk of overfitting through the methods outlined in Section 3.3, Section 3.4, Section 3.5, Section 3.6.1, Section 3.6.2 and Section 3.6.3. First, the color boosting technique (Section 3.3) expands the color distribution of our training data, preventing the model from memorizing a single hue or brightness profile of human traffic controllers (HTCs). Second, *Generating human traffic controller images* (Section 3.5) via our diffusion-based approach introduces novel, previously unseen combinations of styles and poses. Third, the *pasteable region identification* (Section 3.6.1) restricts HTC placement to semantically valid areas, avoiding impractical placements that could bias the model. Fourth, *Instance Size Adjustment Using the Vanishing Point* (Section 3.6.2) incorporates perspective realism, preventing the model from overfitting to a single scale or viewpoint. Lastly, *multi-instance integration* (Section 3.6.3) produces one to three HTCs per image, exposing the model to more complex relationships and reducing its reliance on any single scene composition.

As a result of these combined strategies, the dataset remains both diverse and true to real-world conditions, strengthening the model’s generalization capabilities. In total, the HTC600 dataset comprises 600 real human traffic controller images, 6000 color-boosted real human traffic controller images, 1100 PIDM-generated images, the synthesized training datasets described above, and 135 real-world test images containing at least one human traffic controller instance.

## 4. Experiments

### 4.1. Implementation Details

We conducted experiments using our human traffic controller detection dataset, HTC600, to evaluate the effectiveness of the proposed methodology. We employed YOLOv7 [29] as the detection model. We trained the model on the HTC600 training datasets described in Section 3.6.4, each consisting of 19,840 synthesized training images generated by combining human traffic controller instances with 992 road background images. The key experimental settings and parameters are summarized in Table 1.

For training, we used an RGB input resolution of 640×640. We initially set the learning rate at 10^−2^ and gradually decreased it to 10^−4^ using a cosine decay schedule. We trained the model for 200 to 300 epochs, with early stopping applied if the accuracy did not improve for more than 10 consecutive epochs. We used the Adam optimizer [33], with $\beta_{1}=0.937$ and $\beta_{2}=0.999$, and a weight decay of 5 × 10^−4^. We set the batch size to 32, and the training process required approximately 60 h on a single NVIDIA Titan RTX GPU.

During inference, we applied Non-Maximal Suppression (NMS) with an Intersection over Union (IoU) threshold of 0.45 and a confidence threshold of 0.25 to refine predictions by eliminating overlapping detections. This process allowed the model to accurately detect human traffic controllers in various poses and environments while reducing noisy redundant results.

### 4.2. Quantitative Results

We evaluated the quantitative performance of the proposed method using the HTC600 test dataset, which contains 135 test images with at least one human traffic controller instance. For evaluation, we measured the Average Precision (*AP*) at a 0.5 IoU threshold (*AP*_50_). *AP*_50_ represents the precision at a fixed IoU threshold of 0.5, while *AP* is calculated by averaging precision values at 10 IoU thresholds ranging from 0.5 to 0.95 in increments of 0.05, following the COCO Detection Evaluation Benchmark https://cocodataset.org/#detection-eval (accessed on 16 December 2024).

To highlight the advantages of our approach, we established a baseline by applying a simple cut-and-paste method to generate 19,840 training images using the 600 collected human traffic controller images described in Section 3.1. Starting from this baseline (A), we incrementally applied the proposed enhancements, including realistic transformations (B), color boosting (C), use of PIDM-generated images (D), and color adjustment (E). Table 2 presents the experimental results for the HTC600 test dataset. As shown in Table 2, the baseline approach achieved an *AP*_50_ of 41.0%. By incorporating the proposed methods step-by-step, the performance significantly improved, reaching an *AP*_50_ of 73.9%. This represents a substantial improvement of 32.9%, demonstrating the effectiveness of our approach in enhancing human traffic controller detection accuracy. Each enhancement contributed meaningfully to the overall improvement, underscoring the robustness of the proposed method.

In Table 3, we conduct an ablation study using a state-of-the-art YOLO model. Specifically, we evaluate our method with YOLOv11n (Nano) and YOLOv11s (Small) [34]. The baseline represents method (A) from Table 2, while the proposed corresponds to the integration of methods (A) through (E). The results in Table 3 demonstrate that our proposed method significantly improves detection quality, highlighting its effectiveness in enhancing detection capabilities even when applied to advanced detection networks. The experiments were conducted using the same parameters as the original setup to ensure a fair comparison.

In Table 4, we present an experiment comparing our proposed method with the existing Fallen Person Detection (FPD) method [12]. FPD uses a combination of methods A and E from Table 2, while the proposed corresponds to the integration of methods (A) through (E). Using the FPD method, we generated a training dataset equivalent in size to the HTC600 training dataset, resulting in 19,840 synthetic training images. We conducted an experiment using the HTC600 test dataset, and the results demonstrate that our proposed method outperforms FPD, highlighting its effectiveness in enhancing detection capabilities under challenging conditions.

### 4.3. Qualitative Results

**Figure 8:**
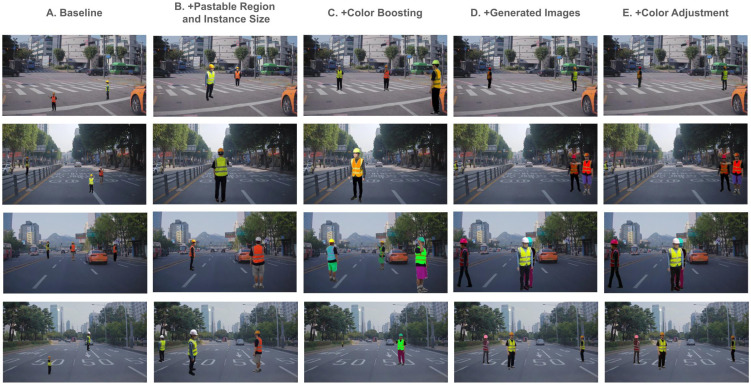
Synthesized training datasets. For each method, we sampled from the same set of background images.

The total synthesized training datasets are shown in Figure 8. In the baseline method (A), the human traffic controllers are often placed in unrealistic locations, such as areas where they would not naturally appear in real-world scenarios. By incorporating pasteable region identification and instance size adjustments (B), we position the human traffic controllers on the road with more realistic scaling, improving visual coherence. Although the color boosting method (C) introduced a wider range of color variations for human traffic controller clothing, the limited number of original human traffic controller images still constrained dataset diversity. By integrating generated images (D), we were able to overcome this limitation by introducing a variety of poses and styles not present in the original dataset, thereby reducing the risk of overfitting. Finally, the color adjustment step (E) harmonized the synthesized human traffic controllers with the road background, creating more realistic composites and improving detection performance in domain-specific scenarios.

**Figure 9:**
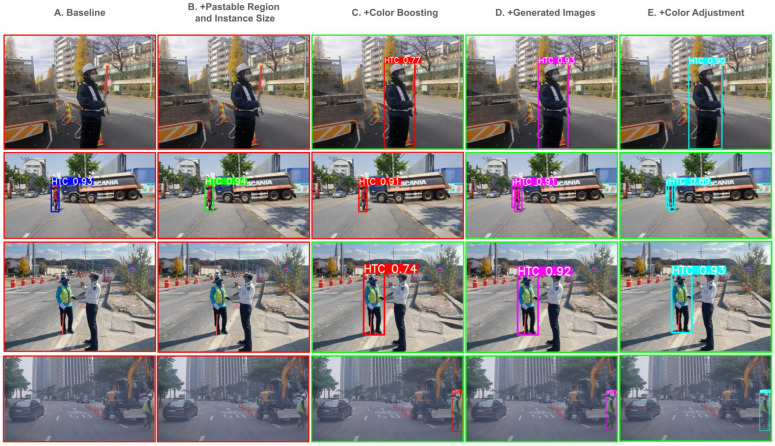
Qualitative comparison of detection results. The columns (A) to (E) represent incremental integration of the proposed methods. Red boxes indicate incorrect detections, while green boxes represent successful detections. From left to right, we observe several red boxes turning into green boxes as additional modules are introduced, indicating more robust and generalized detection.

Figure 9 illustrates the incremental improvements in detection accuracy when training models with methods (A) through (E). As more of our proposed components are added from the left column to the right column, we observe a clear reduction in incorrectly detected results (red) and an increase in correctly detected results (green), highlighting the model’s growing generalizability.

Methods (A) and (B) provide moderate improvements by refining where and how large the human traffic controllers are placed. Method (C) allows the model to detect new styles that were not in the original HTC600 dataset. Then, method (D) enables the model to find poses it missed before, as shown in the second row where two human traffic controllers are finally recognized. Lastly, method (E) aligns the color tones, which further increases the detection confidence.

All these methods help the model handle the variety of clothing, poses, and lighting conditions often seen in real-world settings, highlighting the strength of the proposed approach.

**Figure 10:**
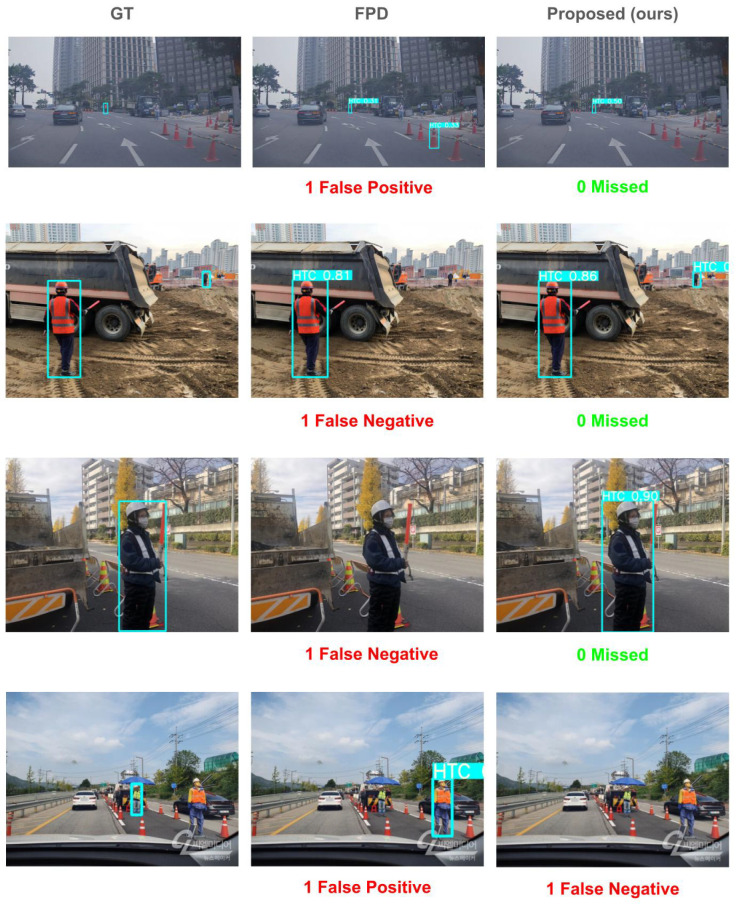
Qualitative results on HTC600 test dataset. From left to right, each row shows the Ground Truth (GT), the method proposed by FPD [12], and Proposed (ours). For each result on the FPD and ours, we denote the number of False Positives and False Negatives.

Figure 10 shows the qualitative results of the proposed methods on the HTC600 test dataset, as well as the results of the FPD [12] method. As shown in Figure 10, our method finds small instances (first and second rows) and effectively demonstrates its ability to detect challenging scenarios. Additionally, in the third row, our method successfully detects a Human Traffic Controller (HTC) that the FPD method fails to identify. Furthermore, in the fourth row, our method avoids false positives by not detecting a mannequin HTC, which the FPD method incorrectly identifies as an actual HTC.

### 4.4. Effect of Daylight

**Figure 11:**
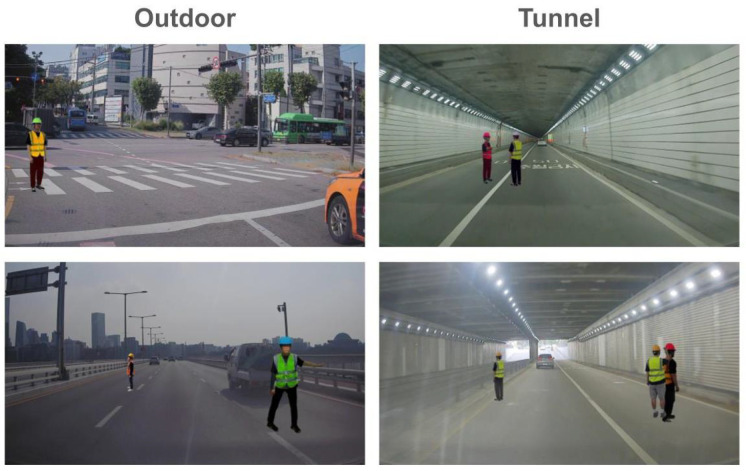
An example of synthesized training dataset in outdoor and tunnel scenes.

In this subsection, we analyze the effect of daylight on the detection performance. We build a hierarchical structure in our HTC600 training set by splitting it into two parts: one is captured under sufficient daylight (i.e., outdoor), and the other one is captured under low daylight (i.e., tunnel). Among 992 road images, 796 images were taken outdoors, and 196 images were taken in a tunnel. Examples of outdoor and tunnel scenes are given in Figure 11.

With this split, we train YOLOv7, and the results for the two different daylight conditions are given in Table 5.

As shown in Table 5, daylight had a marginal impact on performance, with the difference between outdoor and tunnel conditions being approximately 2%. This result suggests that our proposed method is robust to varying daylight conditions and is not significantly affected by changes in illumination. The minimal performance gap demonstrates the adaptability of our approach in handling both sufficient daylight and low-light scenarios effectively, ensuring consistent detection performance across diverse environments.

### 4.5. Practical Implementations

**Figure 12:**
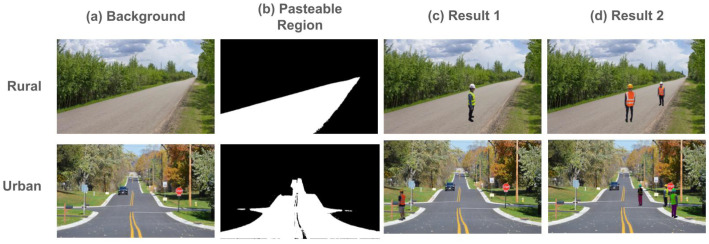
Examples of synthesized images across diverse traffic scenarios. Each row represents a different environment (rural on the top row and urban on the bottom row). Each column shows (**a**) the original background image, (**b**) the pasteable region identified by our method, (**c**) the first synthesized result, and (**d**) the second synthesized result.

In this subsection, we discuss the real-world applications of our proposed model in diverse traffic scenarios, such as rural and urban areas. By leveraging the diverse road background images used in our dataset generation process, the model is equipped to handle a variety of environmental conditions. Specifically, our method identifies pasteable regions within each scene and accurately synthesizes a human traffic controller, ensuring the realism and adaptability of the generated images. Examples of these synthesized images are presented in Figure 12.

As shown in Figure 12, our method can generate well-synthesized images across a variety of traffic scenarios, effectively placing a human traffic controller in different environments without compromising visual realism. By leveraging diverse background images, our approach ensures the adaptable and consistent incorporation of new elements into each scene. This adaptability not only broadens the applicability of the synthesized images for training intelligent transportation systems but also enhances the overall robustness of the model in handling real-world complexities.

### 4.6. Computational Complexity Analysis

To evaluate the capability of YOLOv7 in real-time scenarios, we measured the inference time varying numbers of Human Traffic Controllers (HTCs) using the HTC600 training dataset. The dataset includes scenarios with 1–3 HTCs. As shown in Table 6, our results confirm that the model maintains real-time performance for up to three HTCs.

We further expanded our computational complexity analysis by including both the mean detection time and its standard deviation, as summarized in Table 7. This additional information provides deeper insight into how our model can be deployed in practical real-time scenarios.

In addition, we analyzed the floating point operations (FLOPs) and parameter count of YOLOv7, as summarized in Table 8. This breakdown highlights the computational demands and memory footprint of the model, which are key factors in determining its suitability for real-time deployment.

Overall, the results of these extended analyses confirm that YOLOv7 can operate in real time, making it a viable candidate for real-world applications.

## 5. Conclusions

In this paper, we introduced a novel approach for generating synthetic training data to enhance Human Traffic Controller (HTC) detection at road construction sites. We improved HTC detection performance by combining diffusion-based generation, color boosting, perspective adjustments, and color harmonization. Our method achieved an *AP*_50_ score of 73.9%, surpassing the baseline and demonstrating the effectiveness of synthetic data augmentation in diverse and challenging real-world scenarios. To validate our approach, we developed the HTC600 dataset, which includes real-world images, internet-sourced crops, and synthetic variations. This dataset has been made publicly available to support ongoing research in this domain. While our study effectively addresses key challenges related to data scarcity and appearance variability in HTC detection, it assumes that HTCs control traffic using only their bare hands. This limitation excludes scenarios where HTCs use tools, such as traffic control batons or reflective paddles, which are frequently encountered in real-world settings. To overcome this limitation, future research will focus on expanding the dataset to include tool-assisted traffic control scenarios and developing synthesis methods that enable the generation of HTCs using such tools. These advancements will capture a broader range of real-world conditions and further enhance the robustness and applicability of our detection models.

To make our system safer and avoid misuse in critical traffic conditions, we can explore additional measures in future work. One example is to combine our HTC detection with construction site recognition in [11]. By verifying that an HTC is detected and a genuine construction zone is present, we can prevent unnecessary responses in places where no real construction is happening. This approach makes our system both more reliable and more practical in real-world situations.

By addressing these safety considerations, our study contributes to the advancement of autonomous driving technologies by tackling one of the many corner cases that must be resolved to ensure safe operation in Level 4 autonomous vehicles. Unlike Level 3 systems, which rely on user intervention in challenging scenarios, Level 4 systems operate without any human input, necessitating robust solutions for all potential corner cases. Among these, accurately detecting human traffic controllers during road construction represents a critical scenario for ensuring reliable and safe navigation in dynamic, human-involved road environments. By providing a solution to this specific corner case, our work contributes to the broader goal of enabling autonomous vehicles to navigate safely and reliably across diverse and complex real-world conditions. Furthermore, we emphasize that additional efforts should be made to compress the detector model and reduce its computational load as part of future work.

Overall, our work advances autonomous driving by improving the reliable detection of human traffic controllers, a critical component for ensuring safety in dynamic road environments. By leveraging synthetic data generation, we aim to enhance the safety and reliability of autonomous systems operating in complex and diverse settings.

## Figures and Tables

**Table 1:** Experimental details.

Parameter	Details
Model	YOLOv7
Training Dataset	HTC600 (19,840 synthesized images)
Input Resolution	640 × 640
Learning Rate	Initial: 10^−2^, Final: 10^−4^ (cosine decay)
Training Epochs	200–300 (with early stopping)
Batch Size	32
Optimizer	Adam (lr: 10^−2^, betas: (0.937, 0.999), weight decay: 5 × 10^−4^)
GPU	NVIDIA Titan RTX
Inference Settings	NMS (IoU: 0.45), Confidence Threshold: 0.25
Evaluation Metric	*AP*_50_, *AP* (COCO Detection Benchmark)

**Table 2:** YOLOv7 detection performance on HTC600 test dataset. (Units: Both *AP*_50_ and *AP* are expressed in %, calculated following the COCO detection evaluation benchmark).

Method	*AP*_50_ (%)	*AP* (%)
A. Baseline (Cut and Paste)	41.0	19.6
B. + Pasteable Region and Instance Size	65.7	31.4
C. + Color Boosting	69.6	34.0
D. + Generated Images	72.9	39.2
E. + Color Adjustment	73.9	38.8

**Table 3:** Ablation study with YOLOV11 networks.

Method	YOLOv11n (Nano)		YOLOv11s (Small)	
	*AP*_50_ (%)	*AP* (%)	*AP*_50_ (%)	*AP* (%)
Baseline (A)	51.2	24.5	61.5	35.8
Proposed (A–E)	77.8	53.0	86.8	59.4

**Table 4:** Detection performance comparison on the HTC600 test dataset between FPD [12] and the proposed method.

Method	YOLOv7	
	*AP*_50_ (%)	*AP* (%)
FPD [12]	62.2	36.6
Proposed (A–E)	73.9	38.8

**Table 5:** Results on two different daylight conditions.

Method	YOLOv7	
	*AP*_50_ (%)	*AP* (%)
Outdoor	56.7	31.9
Tunnel	54.3	30.8

**Table 6:** Inference time based on the number of detected Human Traffic Controllers (HTCs).

Number of Detections	Mean Inference Time (ms)	Mean NMS Time (ms)
1	5.87	0.98
2	5.91	0.98
3	5.87	0.98

**Table 7:** Detection time statistics for YOLOv7 (Batch Size = 1).

Metric	Mean Detection Time (ms)	Standard Deviation (ms)
Inference Time	5.9	1.2
NMS Time	1.0	0.1
Total Detection Time	6.9	–

**Table 8:** FLOPs and parameters of YOLOv7.

Model	FLOPs (G)	Number of Parameters (M)
YOLOv7	103.2	36.5

## Data Availability

The raw data supporting the conclusions of this article will be made available by the corresponding author upon reasonable request.
